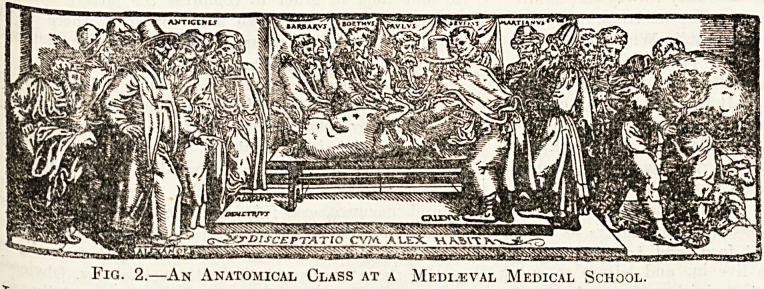# The Surgery of the "Crusades"

**Published:** 1920-12-18

**Authors:** 


### MEDICINE MANY YEARS AGO.

### The Surgery of the "Crusades."

The illustrations which follow are taken from old woodcuts representing medical practice during the Middle Ages. The first block shows the patients consulting a doctor. The "learned leech" stands examining a specimen of urine which he holds in a flask. Other specimens are ranged on the shelves. In the foreground are the patients, two women and a man. Notice, too, the page-boys engaged in a tussle in the foreground!

The second woodcut is a (more or less allegorical) representation of an anatomical lecture at a mediæval medical school. Readers of Longfellow's "Golden Legend" will remember the description which the student gives to his friend of the course at Salern:

> With dissections of the bodies of swine
> As likest the human form divine.

Owing to the scarcity of human bodies, and, in certain places, the laws prohibiting dissections of such bodies, the mediæval doctors were forced to dissect the bodies of animals.

But the work done in the times here represented, although it brought us gradually to the modern surgical marvels of to-day, was preceded by practice unbelievably good dating back to the years of the Crusades. Dr. J. J. Walsh, in his contribution to the Sir William Osler memorial volumes, gives us some remarkable examples of work done at this period of European history. He points out that, in complete contradiction to the common supposition that surgery worthy of the name is an evolution of the last few generations, we have the surgical text-book of Theodoric, who recorded the surgical practice of his father, Hugh of Lucca, a surgeon in one of the earlier Crusades. It is said that this man operated for tumour and abscess inside the cranial cavity! He opened the thorax to drain either pus or fluid, and performed a number of abdominal operations, including the radical cure of hernia with the patient in an exaggerated Trendelenburg position, *i.e.*, head down on a board leaning against the wall!

Fig. 2.—An Anatomical Class at a Mediæval Medical School.

Intestinal anastomoses were made, and wounds in the gut frequently sutured. Metal tubes were employed, and sometimes a portion of the trachea of a lower animal was sewn into position to maintain the intestinal continuity; and it is easy to believe that men who were doing such things were quite capable of surgical intervention for other conditions within the abdomen, and that they must have

Fig. 1.—A Consultation in the Middle Ages.

achieved considerable success, since the record of their work remains after the lapse of seven centuries.

It is extraordinary, too, that in these writings one meets a remarkably clear description of the reason of pus formation, one of the great stumbling-blocks in later mediæval times, and the very expression *per primam intentionem* is a mediæval Latin phrase actually applied to what they sought and did obtain in the healing of the Crusaders' wounds. As we read Theodoric's words more closely, it is obvious that he, even at that time, was on the verge of enunciating the "primary closure of wounds," and the fact is almost startling when we realise how long it has taken the profession actually to

**Medicine Many Years Ago—(*continued*).**

put the method into practice as they did during the past war.

Almost, needless to say, it would have been impossible to do such extensive and deliberate operating without some kind of anæsthetics. When the English poet Middleton, early in the seventeenth century, wrote of "the pities of old surgeons who put their patients to sleep before they cut them," his readers of the generation before our own scarcely knew what to make of Middleton's suggestion of what seems to us *our* anæsthesia. The old Crusader surgeons used a combination of mandragora, opium, wild lettuce, and hyoscyamus for anæsthetic purposes. Tinctures of these were employed and a sponge saturated with them. The technique was to allow this to dry in the sun, and then, having placed it in boiling water, to allow the patient to inhale the steam. There is apparently no doubt that these old-time workers obtained thoroughly efficient, if not always safe, anæsthesia by such means.

In any case, their work could not have been successfully accomplished without thoroughly organised and carefully maintained hospitals. And this, indeed, was the second great anticipation which occurred in the Crusaders' time. We have the story of the organisation of a series of nursing orders, both men and women, whose one purpose was the care of wounded and ailing Crusaders. The famous nursing order of St. John of Jerusalem, whose original purpose was solely to bring in the wounded and to serve in the hospitals, and who came, as a consequence, to be called the Hospitallers, is a typical example.

## Figures and Tables

**Fig. 1. f1:**
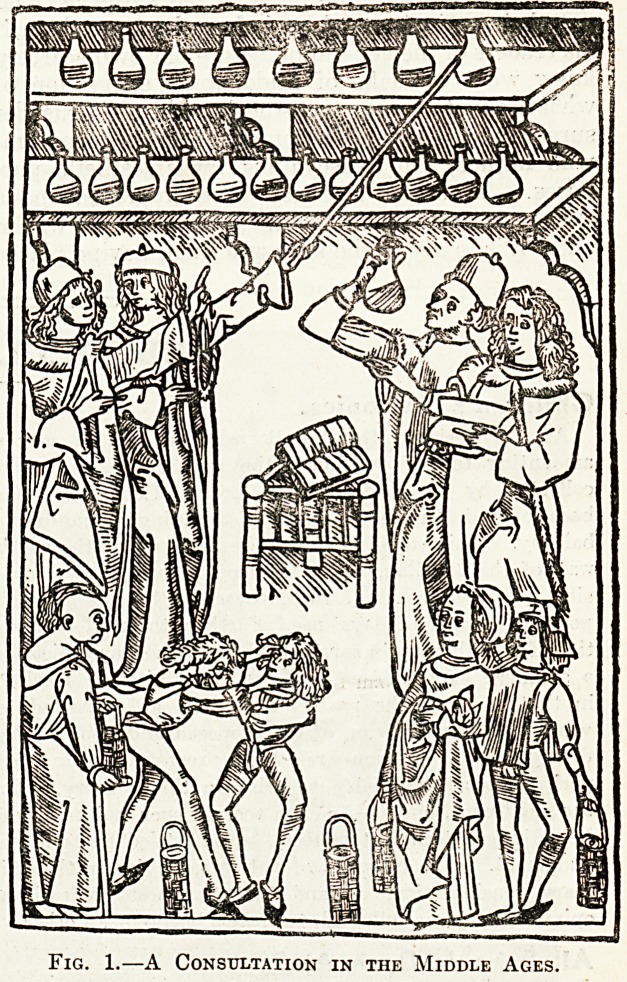


**Fig. 2. f2:**